# A case report of a collision tumor composed of pancreatic ductal adenocarcinoma and peri-pancreatic mucosa-associated lymphoid tissue lymphoma

**DOI:** 10.1186/s12957-023-02981-3

**Published:** 2023-03-28

**Authors:** Ryuji Hirai, Ken-ichi Omae, Mitsuko Yodoya, Syunji Fujie, Masayoshi Fujii, Kazuma Iwata, Kentaro Imanishi, Eisuke Kurihara, Kazuhiro Yoshida, Masaru Jida, Kazuyasu Kobayashi, Yoshiaki Kanaya, Syuichiro Maruyama

**Affiliations:** 1 Department of Surgery, Himeji St. Mary’s Hospital, 650, Nibuno, Himeji, Hyogo 670-0801 Japan; 2 Department of Radiology, Himeji St. Mary’s Hospital, 650, Nibuno, Himeji, Hyogo 670-0801 Japan; 3 Department of Pathology, Himeji St. Mary’s Hospital, 650, Nibuno, Himeji, Hyogo 670-0801 Japan; 4 Department of General Thoracic Surgery and Breast and Endocrinological Surgery, Graduate School of Medicine, Dentistry and Pharmaceutical Science, Okayama University, 2-5-1, Shikata-Cho, Kita-Ku, Okayama City, Okayama 700-8558 Japan

**Keywords:** Collision tumor, Pancreatic cancer, MALT lymphoma

## Abstract

**Background:**

Collision tumors are composed of two distinct tumor components. Collision tumors composed of pancreatic ductal adenocarcinoma and malignant lymphoma occurring in the pancreas have not been previously described in the scientific literature. In this case report, we describe a unique patient with a collision tumor composed of pancreatic ductal adenocarcinoma and peri-pancreatic mucosa-associated lymphoid tissue (MALT) lymphoma occurring in the pancreas.

**Case presentation:**

An 82-year-old woman presented to our hospital complaining of dizziness. Computed tomography (CT) and magnetic resonance imaging (MRI) showed a large lymphoid lesion spreading from the peri-pancreatic tissue heading to the hepatic hilar plate, involving the hepatoduodenal ligament and the entire duodenum, also showing a hard tumor in the pancreas head. We performed echo-guided needle biopsies for each tumor and diagnosed a collision tumor composed of pancreatic ductal adenocarcinoma and low-grade B cell lymphoma. The patient underwent pancreaticoduodenectomy. The resected specimen showed an elastic hard tumor, 90 × 75 mm in size, located in the pancreatic head, and a whitish-yellow hard tumor involving the lower bile duct, 31 mm in size, located in the center of the pancreatic head. Pathological and immunohistochemical examination proved that pancreatic ductal adenocarcinoma and MALT lymphoma originating from the peri-pancreatic head collided in the pancreatic head.

**Conclusions:**

To best of our knowledge, this is the first report of a surgically resected collision tumor of pancreatic ductal adenocarcinoma and MALT lymphoma originating from the peri-pancreatic head. A needle biopsy is useful when inconsistent findings are observed on diagnostic CT and MRI of tumor lesions since there is the possibility of a collision tumor.

## Background

Collision tumors are composed of two distinct tumor components. Most tumors are described in neurosurgery, dermatology, and urology. In visceral surgery, most collision tumors are noted in the stomach, such as the gastrointestinal stromal tumor (GIST) and adenocarcinoma. Collision tumors composed of pancreatic ductal adenocarcinoma and malignant lymphoma occurring in the pancreas have not been previously described in the scientific literature. In this case report, we describe a unique patient with a collision tumor composed of pancreatic ductal adenocarcinoma and peri-pancreatic mucosa-associated lymphoid tissue (MALT) lymphoma occurring in the pancreas.

## Case presentation

An 82-year-old woman visited the emergency room of Himeji St. Mary’s Hospital complaining of dizziness. She had a gastric ulcer and had undergone distal gastrectomy 40 years previously. There were no abnormal findings on head computed tomography (CT) scanning and otorhinological examination, excluding blood examination showing an increase in hepatobiliary enzymes level, CA 19–9, and DUPAN-II (Table 1). After admission, the sIL-2 receptor was revealed to be high at 1710 U/mL.

**Table 1:**
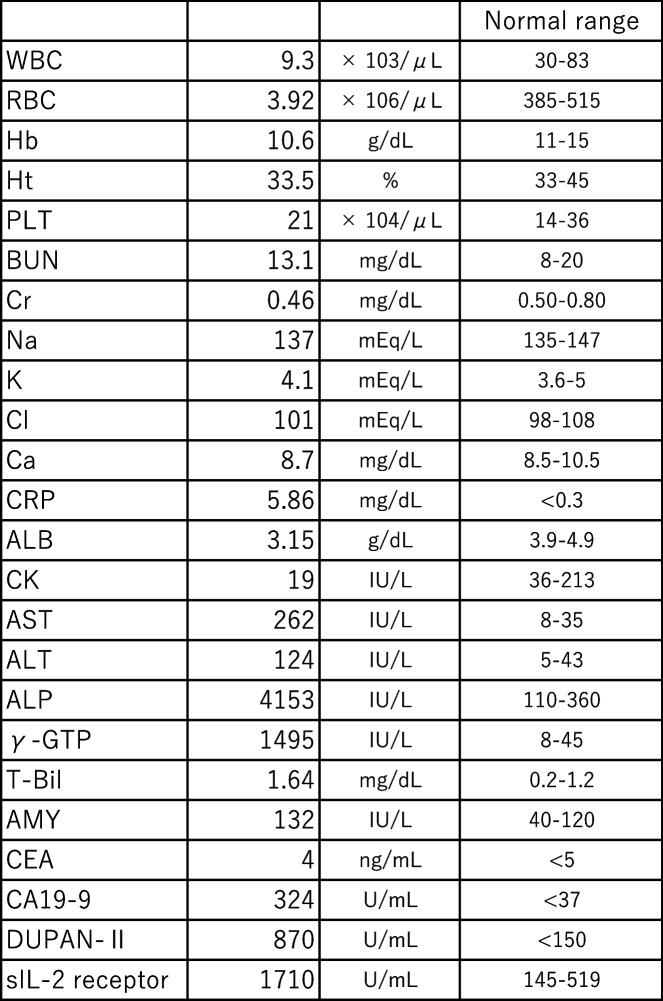
Laboratory data on admission

**Fig. 1:**
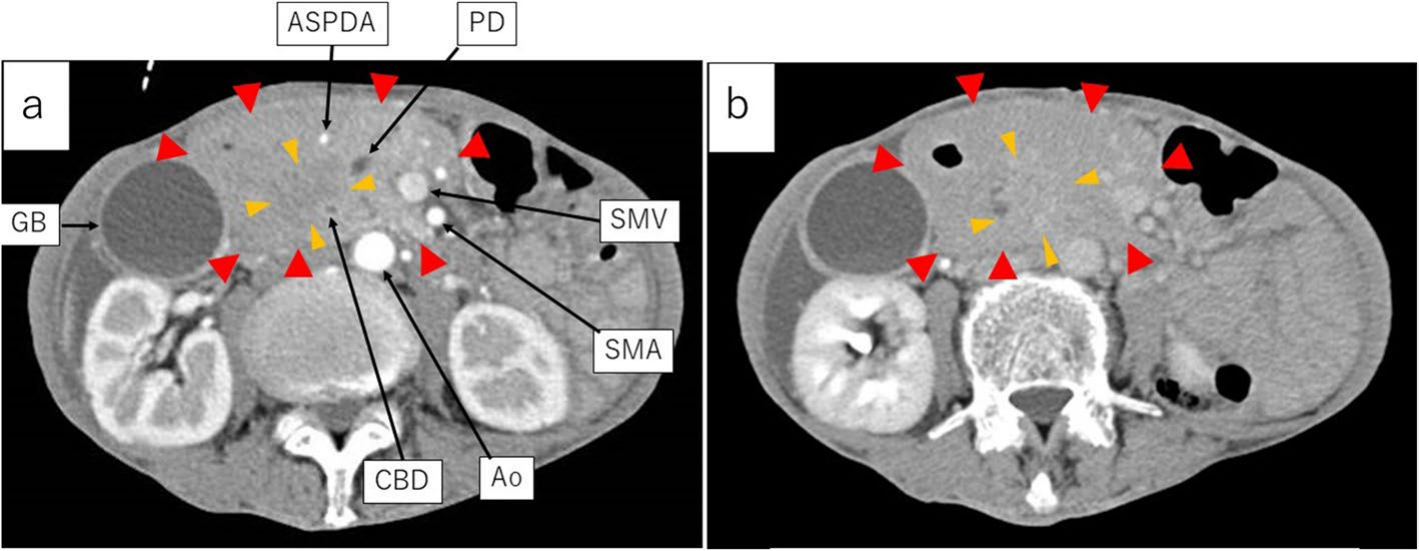
Contrast-enhanced CT (axial view); **a** Early phase. **b** Late phase. Contrast-enhanced CT revealed that the peripheral part had a slightly low density in the early phase and homogeneous isodensity in the late phase. However, the central part revealed a lower density in the early phase and a heterogeneous high density in the late phase. Ao aorta, ASPDA anterosuperior pancreaticoduodenal artery, CBD common bile duct, Du duodenum, GB gall bladder, SMA superior mesenteric artery, SMV superior mesenteric vein, PD pancreatic duct, PV portal vein

**Fig. 2:**
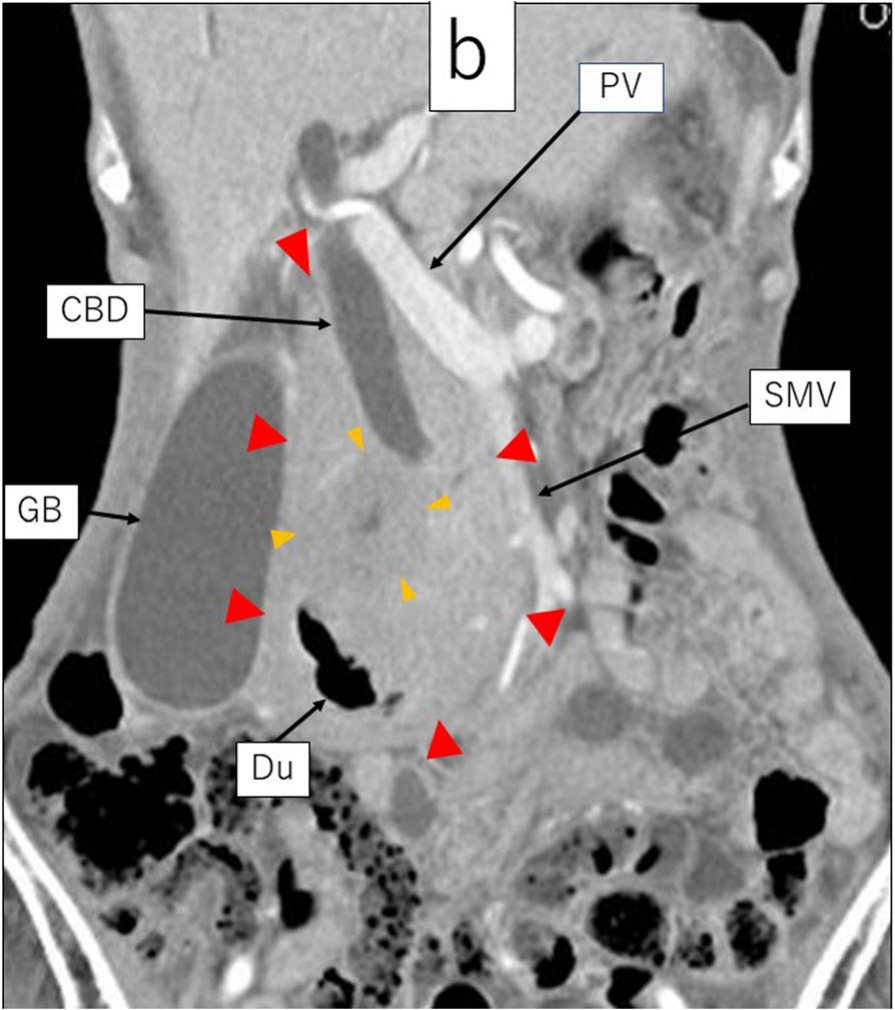
The early phase of contrast-enhanced CT (coronal view); A large homogeneous low-density lesion (  ) spread widely from the peri-pancreatic head to the hepatic hilar plate, the hepatoduodenal ligament, duodenum, and the root of the mesenteric artery and vein. The intrahepatic duct and the common hepatic duct were dilated. The lower bile duct was involved and obstructed by the central part (  ) which showed lower density than the peripheral part. Ao aorta, ASPDA anterosuperior pancreaticoduodenal artery, CBD common bile duct, Du duodenum, GB gall bladder, SMA superior mesenteric artery, SMV superior mesenteric vein, PD pancreatic duct, PV portal vein

Abdominal contrast-enhanced CT showed a large homogeneous low-density lesion, 13 × 9 × 5 cm in size, widely spreading from the peri-pancreatic head to the hepatic hilus, the hepatoduodenal ligament, the duodenum, and root of the mesenteric vessels. Intrahepatic duct and common hepatic duct were dilated, but the lower bile duct was obstructed by the lesion’s central part. The main pancreatic duct of the pancreatic head was also obstructed by the lesion’s central part. The portal vein and superior mesenteric vein went through the lesion’s peripheral part but were neither involved nor narrowed. The duodenal wall was thickened, but the duodenum lumen was not narrowed. The peripheral part had a slightly low density in the early phase and homogeneous isodensity in the late phase. However, the central part revealed a lower density in the early phase and a heterogeneous high density in the late phase (Figs. 1 and 2).

**Fig. 3:**
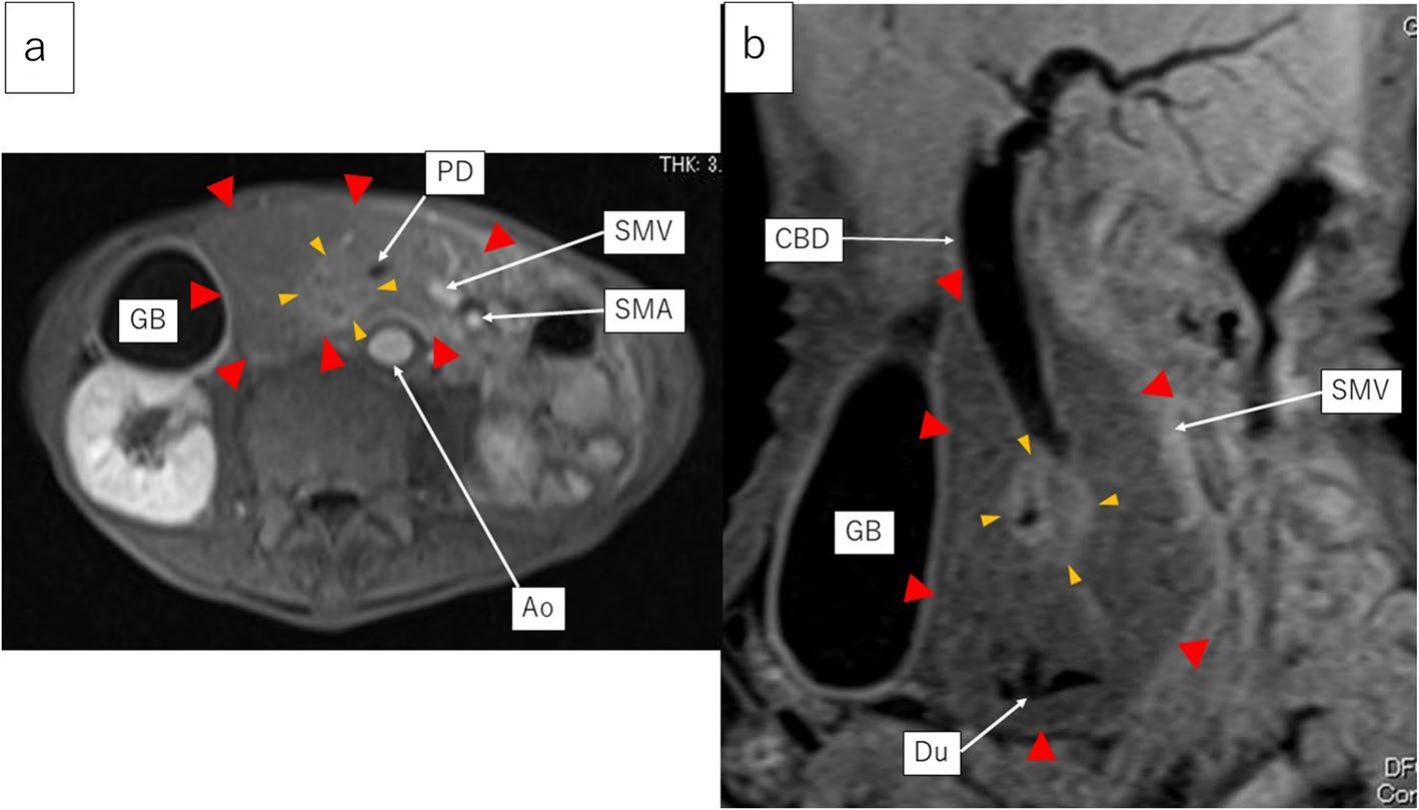
The late phase of contrast-enhanced MRI; **a** Axial view. **b** Coronal view. The peripheral part of the lesion had a low signal, while its central part had a high signal. The lower bile duct and the main pancreatic duct were involved and obstructed by the central part of the lesion. However, the upper and middle bile duct, superior mesenteric vein, duodenum, and gall bladder were not involved by the peripheral part of the lesion. Ao aorta, CBD common bile duct, Du duodenum, GB gall bladder, SMA superior mesenteric artery, SMV superior mesenteric vein, PD pancreatic duct

Contrast-enhanced MRI showed that the peripheral part had a low signal in the early phase and a slightly low signal in the late phase. In contrast, the central part revealed a low signal in the early phase and a heterogeneous high signal in the late phase (Fig. 3).

**Fig. 4:**
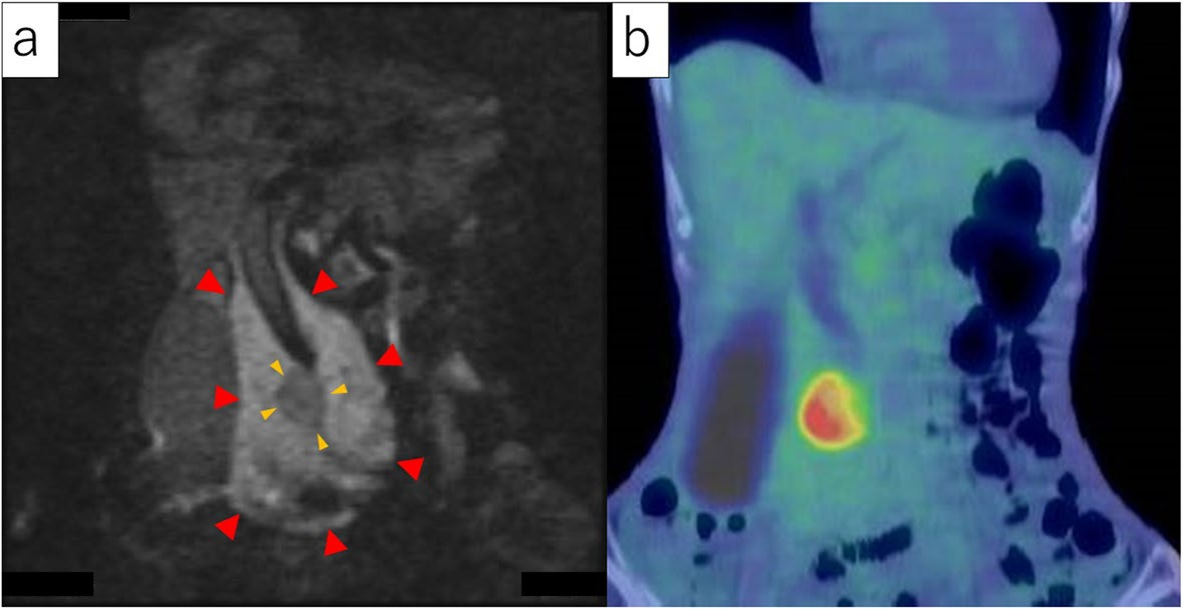
**a** Diffusion-weighted images of MRI. The peripheral part had a very high signal, while the central part had a relatively lower signal. **b** Positron emission tomography. A strong accumulation of fluorodeoxyglucose was seen in the central part, suggesting a high-grade malignant tumor

Diffusion-weighted imaging (DWI, *b* = 800 s/mm^2^) showed a very high signal in the peripheral part and a relatively lower signal in the central part (Fig. 4a). The peripheral part revealed high-cell density and was considered as malignant lymphoma due to the markedly low diffusion. However, the central part seemed to be a different kind of tumor from the peripheral part. Positron emission tomography (PET) showed a strong accumulation of fluorodeoxyglucose (FDG) on the central part, suggesting a high-grade malignant tumor (Fig. 4b).

### Preoperative imaging diagnosis

**Fig. 5:**
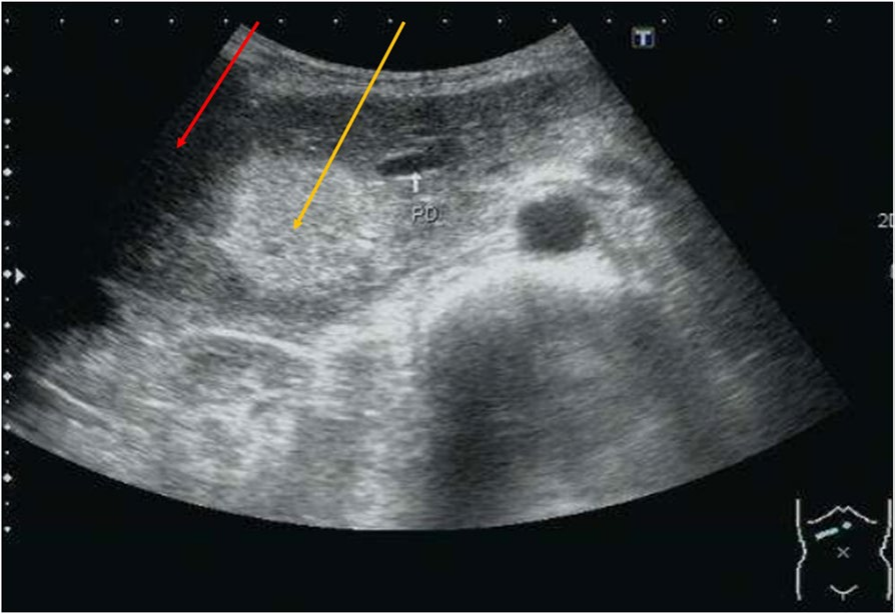
Ultrasound image. Echo-guided needle biopsies were performed at the high echoic central part (  ) and low echoic peripheral part (  ), respectively

The peripheral part was certainly malignant lymphoma, and the central part seemed to be a malignant tumor originating from the epithelium, such as pancreatic carcinoma. Therefore, we considered that two histologically different tumors collided in the pancreas head. For a definite diagnosis, percutaneous ultrasound-guided needle biopsies were performed at the hyperechoic central lesion and hypoechoic peripheral lesion (Fig. 5).

**Fig. 6:**
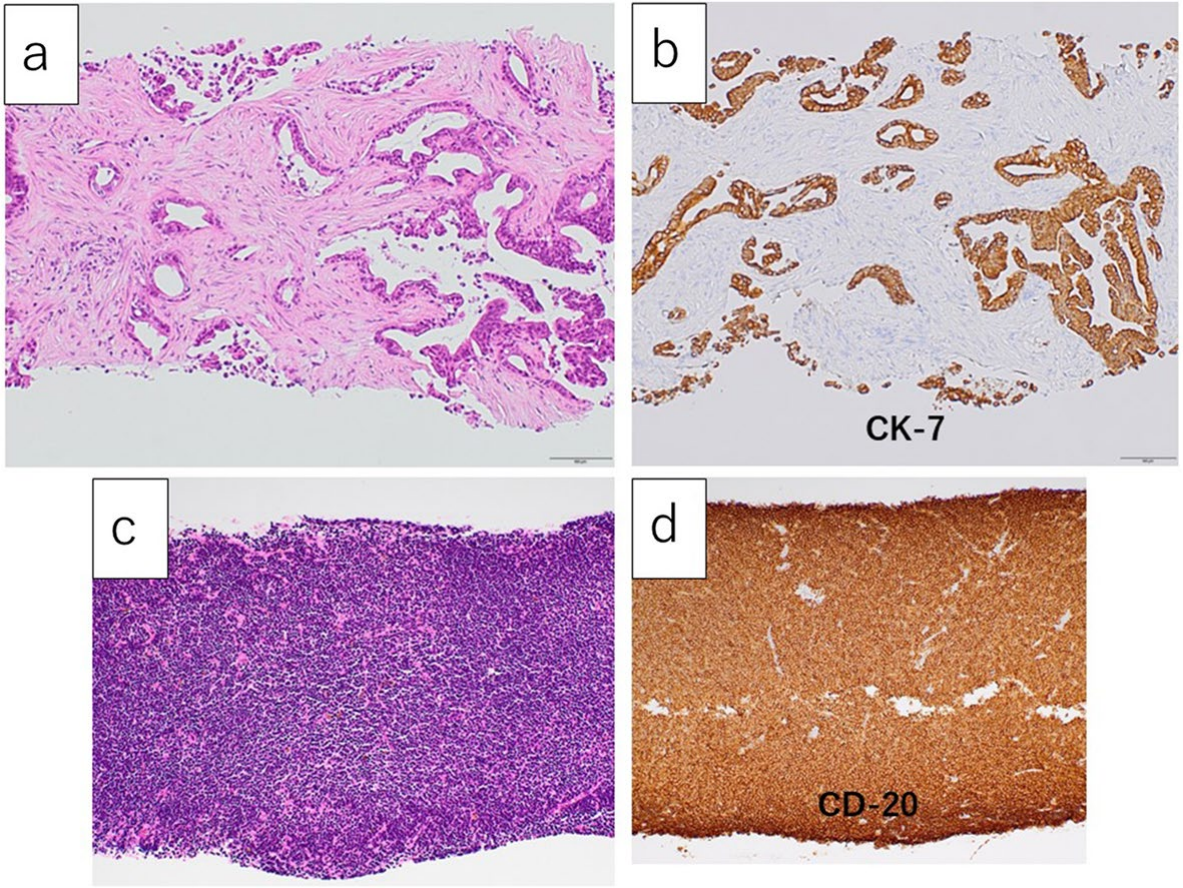
Microscopic findings of the needle biopsy specimens. The central part (**a** H.E. staining, **b** CK7 staining, × 40) and the peripheral part (**c** H.E. staining, **d** CD20 staining, × 40). The central part was diagnosed as an adenocarcinoma positive for CK7 and the peripheral part was diagnosed as a low-grade B cell lymphoma positive for CD20, characterized by diffuse proliferation of small lymphoid cells

Histopathological findings revealed that the central lesion was diagnosed as an adenocarcinoma positive for CK7, negative for CK 20 and CDX2, and with a very high Ki-67 labeling index (Fig. 6a). In contrast, the peripheral lesion was diagnosed as a low-grade B cell lymphoma as CD20-positive, CD3-negative, CD10-negative, CD5-negative, CyclinD1-negative, BCL2-positive, BCL6-negative, cytokeratin-negative, and with a low-labeling index by Ki-67 (Fig. 6b).

We preoperatively presumed that this lesion was a collision tumor composed of pancreatic head carcinoma, T2N0M0, Stage IB (UICC ver.8), and low-grade B cell lymphoma, StageIIE (the Lugano classification).

### Treatment strategy

Since it was clear that the pancreatic carcinoma would determine the prognosis of this patient, we decided to give priority to treating the carcinoma. The preoperative diagnosis indicated that the pancreatic carcinoma was T2N0M0, stage IB (UICC), which prompted us to decide that neoadjuvant chemotherapy was not a mandatory choice. We devised a strategy in which pancreaticoduodenectomy (PD) would be performed first, and once the patient was ready for chemotherapy after post-operative recovery, the malignant lymphoma would be treated with chemotherapy. Further, if the pancreatic carcinoma relapsed, it would be treated with chemotherapy. However, due to the advanced age of the patient, any chemotherapy carried a high risk. It was thus decided that careful consideration must be given as to whether or not to conduct chemotherapy. As for the operation, we determined the patient to be operable even with advanced age and low weight and decided to perform PD.

### Surgical procedure

Previously, the patient underwent distal gastrectomy and Billroth II gastro-jejunostomy; therefore, we cut the afferent loop near the anastomotic part. After Kocher maneuver, we palpated a fist-sized tumor on the pancreas head. When detaching the pancreatic head from the transverse mesocolon, we found thickening of the transverse mesocolon along the right branch of the middle colic artery, and it was indicative of progressive lymphoma. The lymphoma tissue around the middle colic artery was incompletely removed because it appeared impossible to resect diffusely spread lymphoma fully and because the residual lymphoma could be treated with chemotherapy. Intraoperative echography demonstrated hypoechoic to isoechoic lymphoma in the pancreatic head, with an unclear border between the lymphoma in the pancreatic head and the non-lymphoma area in the pancreatic body and tail. However, the central part of the lesion, which seemed to be adenocarcinoma, was hyperechoic and had a clear border; therefore, the pancreas was resected once directly above the portal vein. Since intraoperative rapid pathological examination revealed that lymphoma tissue was present in at least half of the area of the resected pancreatic stump cut surface, an additional 3 cm of the pancreas was resected, and a negative stump was confirmed. The nerve plexus around the superior mesenteric artery (SMA) was not resected because there was sufficient distance between the pancreatic cancer area and the SMA.

**Fig. 7:**
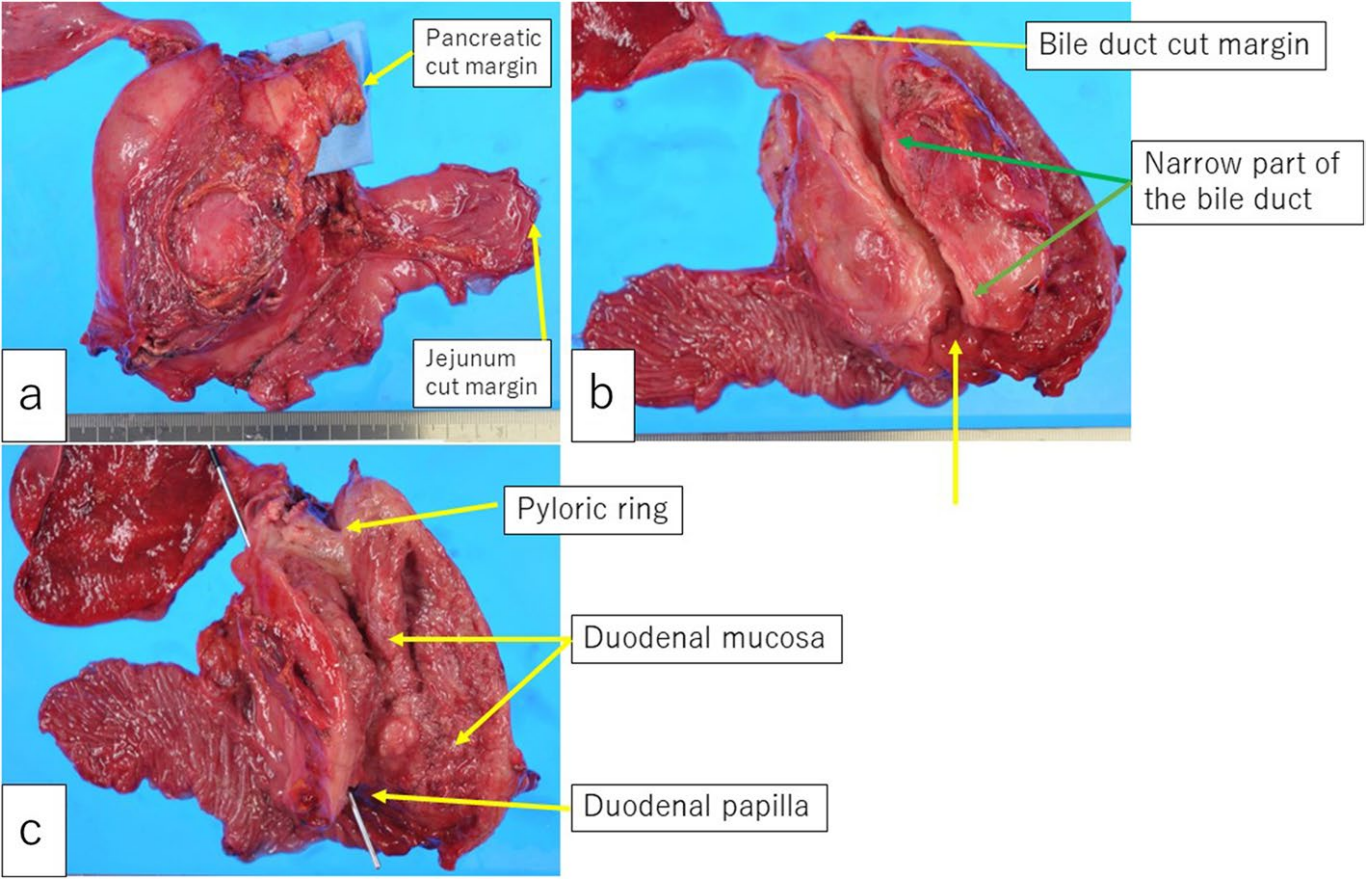
Macroscopic findings of the resected specimen; **a** Front view. **b** View of the back of the pancreas head. **c** View of the duodenal lumen. Elastic hard tumor, 90 × 75 mm in size, located in the pancreatic head (**a**). The lower bile duct was involved with the tumor, showing the 40-mm long narrow part (**b**). The duodenal lumen showed a diffusely elevated lesion with irregular thickness on the second duodenal segment (**c**)

The resected specimen showed an elastic hard tumor, 90 × 75 mm in size, located in the pancreatic head (Fig. 7a). The lower bile duct was involved with the tumor, showing a narrow part of 40 mm in length (Fig. 7b). The second part of the duodenum had a diffusely elevated mass with irregular thickness on the mucosal layer (Fig. 7c).

**Fig. 8:**
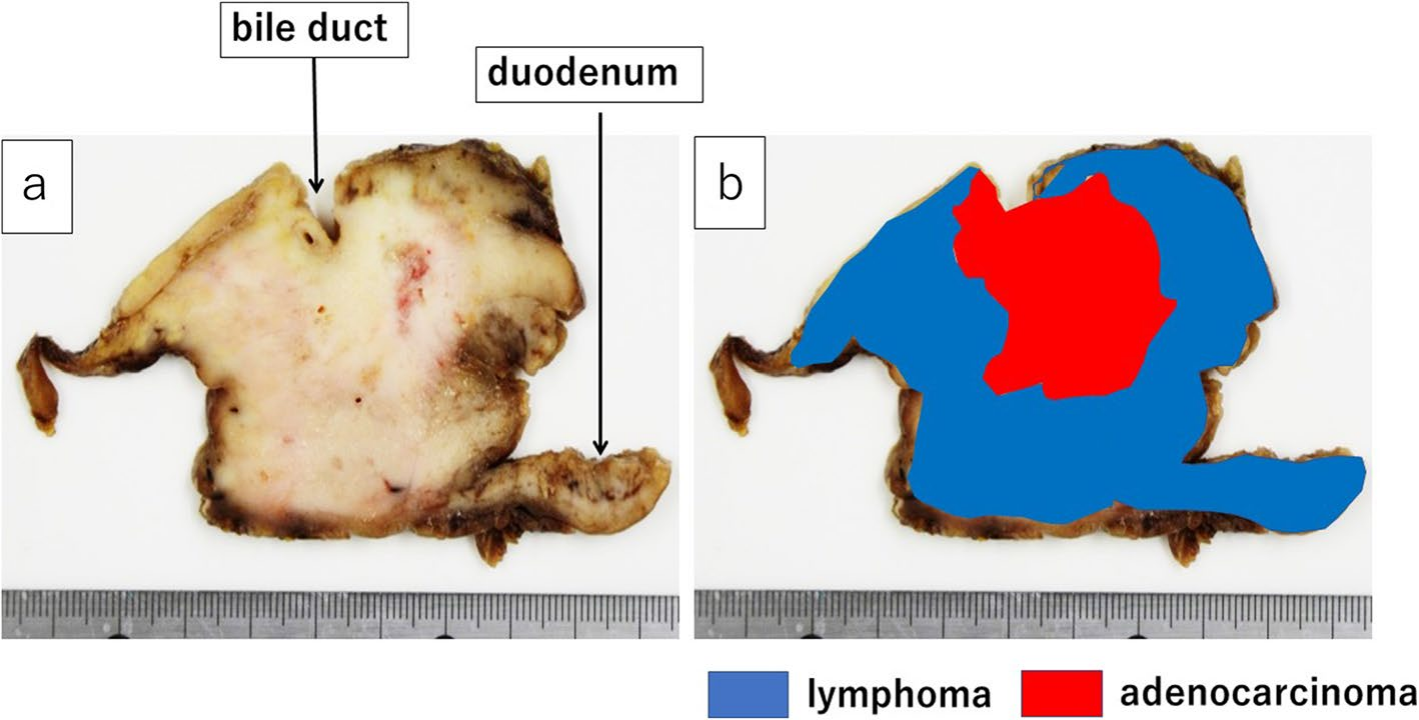
**a** The cut surface of the formalin-fixed specimen. The whitish-hard tumor with an irregular border on the central part of the lesion, including the necrotic part inside, involving the bile duct, was considered a pancreatic adenocarcinoma, while the whitish-yellow peripheral tumor was considered a malignant lymphoma. **b** Schema of the cut surface. The red area, representing the adenocarcinoma, was surrounded by the blue area, representing malignant lymphoma

The cut surface of the formalin-fixed specimen showed a whitish hard central lesion, including the necrotic part inside, with an irregular border, involving the bile duct, was considered the pancreatic carcinoma, while the whitish-yellow peripheral lesion was considered malignant lymphoma (Fig. 8).

**Fig. 9:**
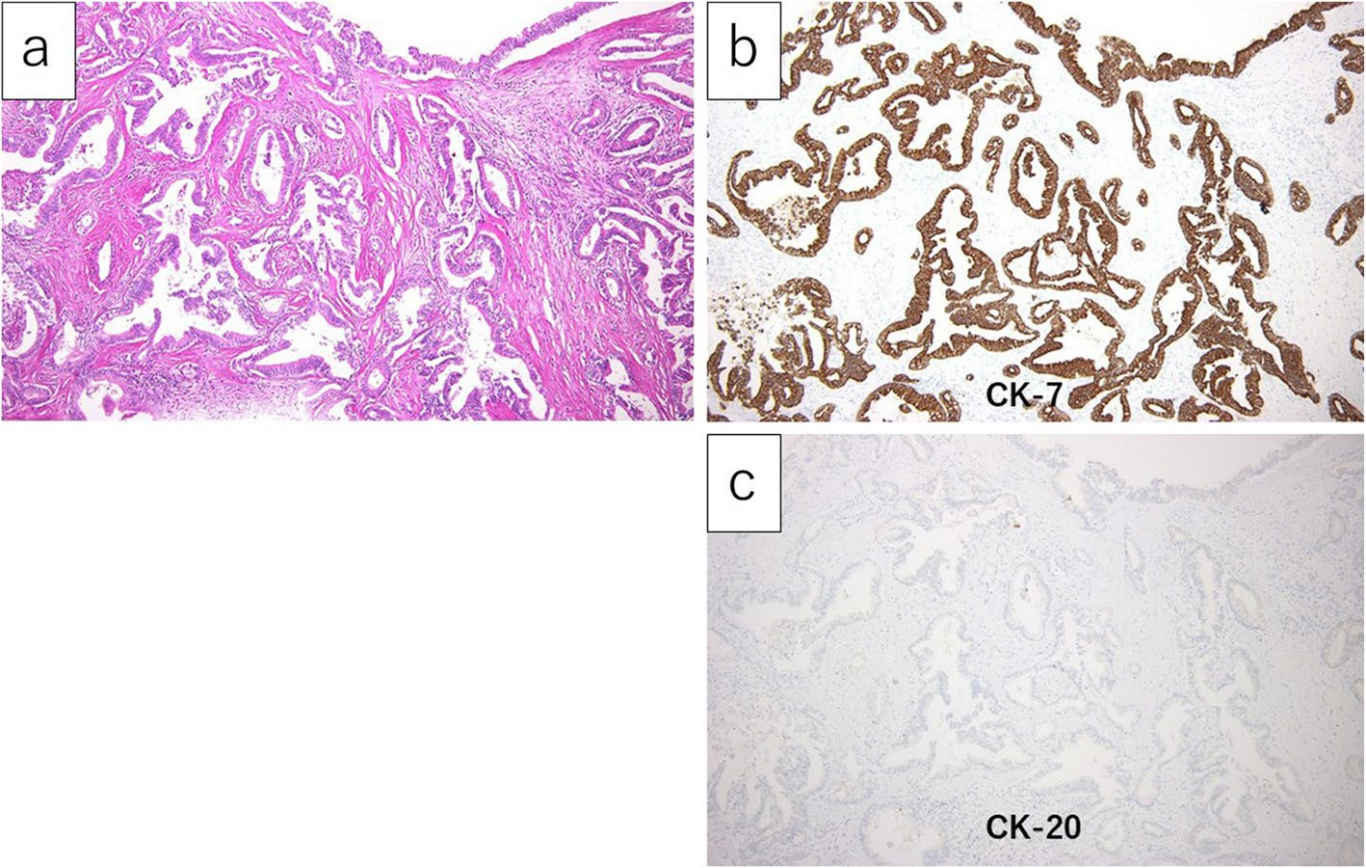
The microscopic findings of the central part of the resected specimen. **a** The central part was composed of mixed well-to-moderately differentiated glands and poorly differentiated solid cell sheets (× 100). **b**, **c** On immunohistochemical examination, the tumor cells were positive for CK7 (**b** × 100) and negative for CK20 (**c** × 100)

Microscopic examination showed that the central lesion, 31 × 30 × 28 mm in size, invading the common bile duct in the pancreatic head, comprised an intermix of well-to-moderately differentiated glands and poorly differentiated solid cell sheets (Fig. 9a). The glands contained luminal and intracellular mucins. Immunohistochemical staining revealed that the tumor cells were diffusely positive for CK7 (Fig. 9b) and negative for CK20 (Fig. 9c).

**Fig. 10:**
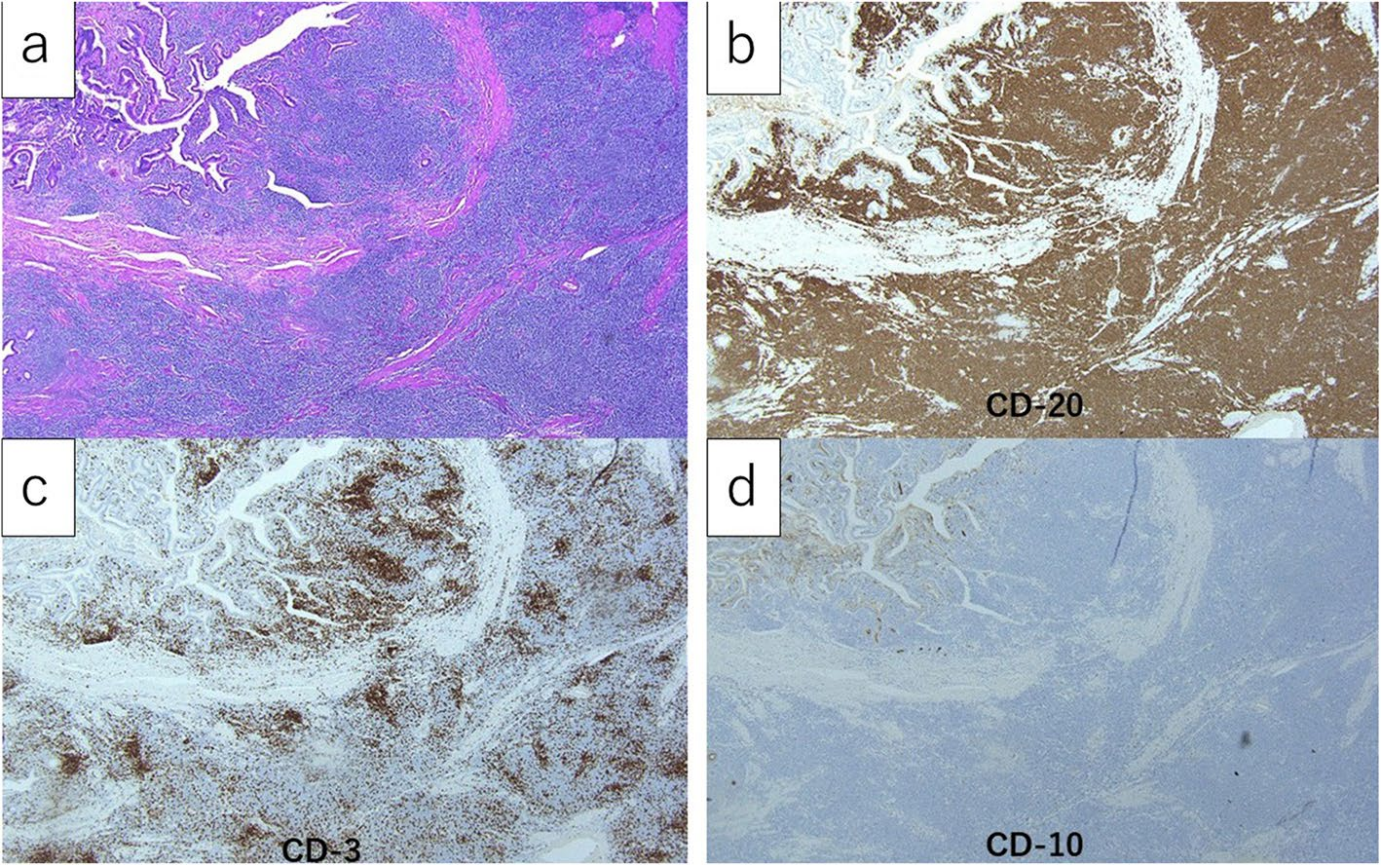
The microscopic findings of the peripheral part of the resected specimen. **a** The peripheral part showed diffuse proliferation of small lymphoid cells (× 40). **b–d** Immunohistochemically, the infiltrating lymphoid cells were diffusely and strongly positive for CD20 (**b** × 40) and negative for CD3 (**c** × 40) and CD10 (**d** × 40)

The central lesion was finally diagnosed as a pancreatic ductal adenocarcinoma, R0, pT2pN0M0, pStageIB (staged by UICC ver.8). On the other hand, the peripheral lesion showed diffuse proliferation of small lymphoid cells (Fig. 10a), replacing the entire duodenal wall, involving the mucosa, submucosa, and muscular propria, extending interminably into the pancreatic glands and stroma, and further infiltrating into the adjacent peritoneum and retroperitoneum. Some cells showed Dutscher bodies or plasma cell differentiation, but monocytoid cells were not present. Immunohistochemical staining revealed that infiltrating lymphoid cells were diffusely positive for CD20 (Fig. 10b) and BCL2, while the number of CD3-positive cells was very low (Fig. 10c). These CD20-positive cells were negative for CD5, cyclin D1, SOX11, CD10, and BCL6, with a low level of Ki67-labeling index (Fig. 10d).

**Fig. 11:**
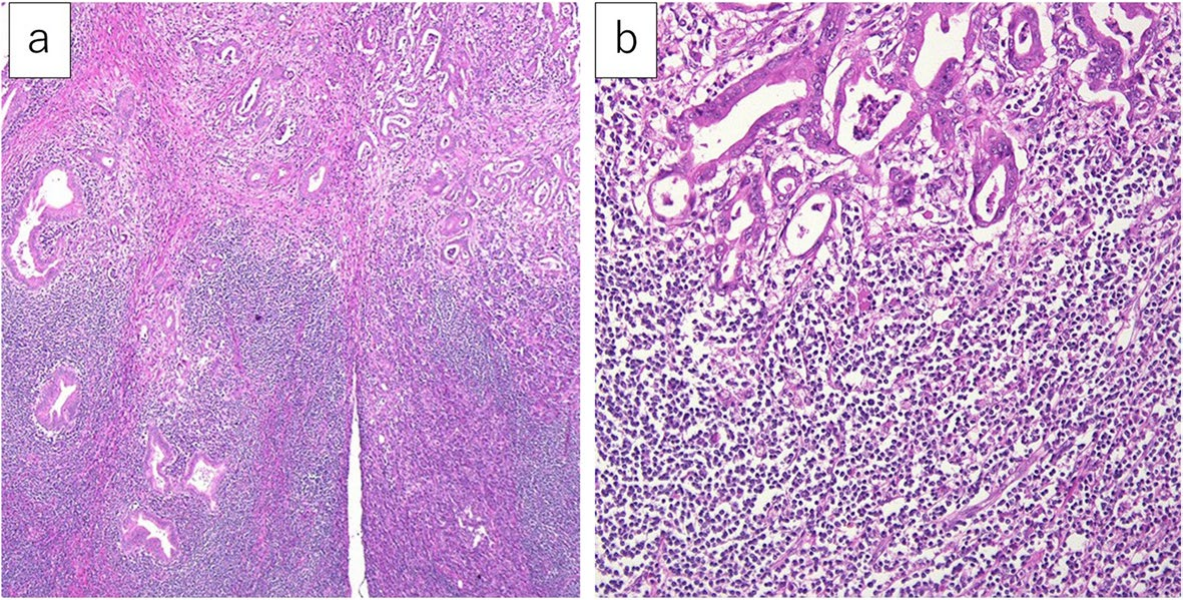
The microscopic findings of the resected specimen. **a** Low power field. **b** High power field. The pancreatic ductal adenocarcinoma (upper part) and the MALT lymphoma (lower part) collided in the pancreatic head (**a**, × 100; **b**, × 400)

Lymphoepithelial lesions were focally seen in the duodenal mucosa, while a few were observed in the pancreas. Thus, the origin of the lymphoma remains unclear. The peripheral lesion was diagnosed as an extranodal marginal zone lymphoma of mucosa-associated lymphoid tissue (MALT) lymphoma, the Lugano classification StageIIE. In conclusion, we diagnosed a collision tumor composed of pancreatic ductal adenocarcinoma and MALT lymphoma, mainly involving the peripheral pancreatic head and duodenum (Fig. 11).

### Postoperative course

A pancreatic fistula occurred after the surgery. Several weeks after the management of the pancreatic fistula, she was discharged from the hospital. Five months after the surgery, she was admitted to the hospital due to a compression fracture of the lumbar vertebra and malnutrition. Abdominal computed tomography (CT) revealed a metastatic lesion on the abdominal wall and pleural effusion. After a 1-month stay, she was transferred to another hospital for further rehabilitation. Several days later, she suddenly developed respiratory failure and died approximately 6 months after the surgery.

## Discussion

This is the first case report of a collision tumor composed of pancreatic ductal adenocarcinoma and MALT lymphoma occurring in the pancreas. A PubMed search of “collision tumor” showed 1742 studies to date. Most tumors are described in neurosurgery, dermatology, and urology. In visceral surgery, most collision tumors are described in the stomach such as GIST and adenocarcinoma. In addition, a PubMed search for “collision tumor” and “adenocarcinoma” and “malignant lymphoma” yielded 64 hits, of which 43 cases were eligible. The most common site was the stomach (*n* = 22), followed by the colon (*n* = 11), lymph nodes (*n* = 4), mammary gland (*n* = 3), liver (*n* = 2), and duodenal papilla (*n* = 1). There is no eligible case colliding in the pancreas. Dasanu et al. reported a collision tumor of pancreatic adenocarcinoma and mantle cell lymphoma, but the collided tumor was found in the liver and composed of metastatic liver cancer from pancreatic adenocarcinoma and mantle cell lymphoma diagnosed Ann Arbour stageIVB [1]. Therefore, this case was not eligible as a collision tumor occurring in the pancreas.

Furthermore, a PubMed search on “pancreatic ductal adenocarcinoma” and “collision tumor” revealed that there are only nine publications about collision tumor consisting of pancreatic ductal adenocarcinoma and other tumors colliding in the pancreas (Table 2).

**Table 2:**
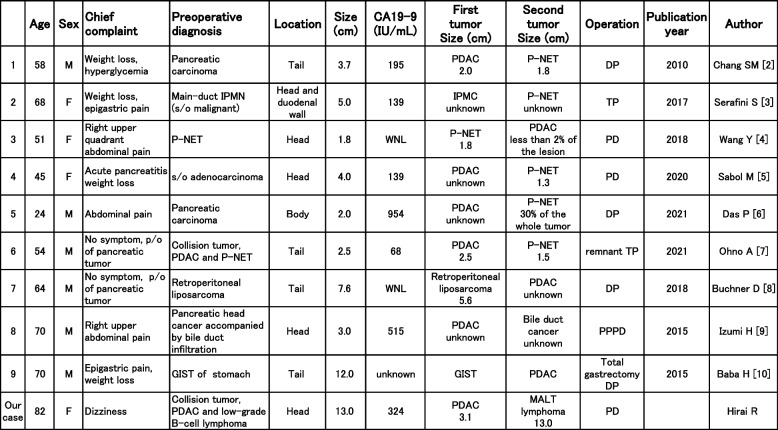
Collision tumors composed of pancreatic ductal adenocarcinoma and other tumors colliding in the pancreas

Six of nine are neuroendocrine tumors of the pancreas [2–7], and others are comprised of a retroperitoneal liposarcoma [8], a bile duct carcinoma [9], and a gastric stromal tumor [10]. The frequency of collision with neuroendocrine tumors might be explained by the fact that neuroendocrine tumors are frequent secondary solid tumors arising from the pancreas. The chief complaints in these nine cases were abdominal pain (*n* = 4), weight loss (*n* = 4), and others (*n* = 1). The most common preoperative diagnosis was suspected pancreatic carcinoma (*n* = 5), and others were pancreatic neuro-endocrine tumor (P-NET), GIST, and liposarcoma (*n* = 1 each). Therefore, only one case had a preoperative diagnosis of collision tumor. The disposition of the site was the head (*n* = 4), body (*n* = 1), and tail (*n* = 4). CA19-9 was elevated in six cases. All patients underwent pancreatectomy: pancreaticoduodenectomy (*n* = 3), distal pancreatectomy (*n* = 5), and total pancreatectomy (*n* = 1). A collision tumor was diagnosed after surgery and was found by chance in eight other cases than the one diagnosed preoperatively. On the other hand, a collision tumor with malignant lymphoma in the pancreas has not been reported previously; therefore, the present case is the first case of a collision tumor composed of pancreatic ductal adenocarcinoma and malignant lymphoma occurring in the pancreas.

With regard to image diagnosis, each tumor exhibited typical findings. Contrast-enhanced CT revealed that the peripheral part had a slightly low density in the early phase and homogeneous iso-density in the late phase. However, the central part revealed a lower density in the early phase and a heterogeneous high density in the late phase. Thus, each tumor presented the typical features of malignant lymphoma and pancreatic ductal adenocarcinoma, respectively. Contrast-enhanced MRI showed that the peripheral part had a low signal in the early phase and a slightly low signal in the late phase. In contrast, the central part revealed a low signal in the early phase and a heterogeneous high signal in the late phase. Moreover, contrast-enhanced MRI demonstrated typical patterns for each tumor. DWI (*b* = 800 s/mm^2^) showed a very high signal in the peripheral part and a relatively lower signal in the central part. The peripheral part revealed high-cell density and was considered as malignant lymphoma due to the markedly low diffusion. However, the central part seemed to be a different kind of tumor from the peripheral part, indicating a pancreatic ductal adenocarcinoma. Furthermore, the portal vein and SMA in the peripheral lesion were uninvolved and penetrating the tumor, indicating typical findings of malignant lymphoma. Leite et al. also reported that the envelopment of adjacent vessels without evidence of obstruction is suggestive of lymphoma [11]. In contrast, the main pancreatic duct and common bile duct were involved and obstructed, indicating typical findings of pancreatic ductal adenocarcinoma. Moreover, high uptake on the central part by FDG-PET suggested pancreatic ductal adenocarcinoma.

MALT lymphoma arises from the mucosa-associated lymphoid tissue of the gastrointestinal tract, salivary glands, lungs, and thyroid glands. According to Tanaka et al., the gastrointestinal tract tumors mostly arise from the stomach (76.9%) and colon/rectum (16.5%), but rarely arise in the duodenum (3.6%), small intestine (2.4%), and esophagus (0.6%) [12]. This disease is a lymphoid tumor with low-grade malignancy, representing characteristic histopathological features, such as centrocyte-like cells with positive B cell surface markers, lymphoepithelial lesions, lymph-follicular proliferation, and infiltration of plasma cells [13]. Differential diagnosis for marginal zone B cell lymphoma mainly depends on immunohistochemistry, including at least CD20, CD10, CD5, CD23, cyclin D1, immunoglobulin D, and SOX11 [14]. In the present case, immunohistochemical staining revealed that infiltrating lymphoid cells were diffusely positive for CD20 and BCL2, while CD3-positive cells were negative. These CD20-positive cells were negative for CD5, cyclin D1, SOX11, CD10, and BCL6, with a low level of Ki67-labeling index. Finally, the lesion was diagnosed as a MALT lymphoma.

Primary pancreatic lymphoma (PPL) is a rare disease [15], encountered in only 0.5% of patients undergoing endoscopic ultrasound-guided fine needle aspiration of solid pancreatic masses [16]. Behrns et al. defined the diagnostic criteria of PPL as follows: mass predominantly located within the pancreas, with grossly involved lymph nodes confined to the peripancreatic region, no palpable superficial lymphadenopathy, no hepatic or splenic involvement, no mediastinal nodal enlargement on chest radiography, and a normal white blood cell count [17]. In our case, the tumor had spread widely, mainly to the pancreatic head, hepatic portal region on the head side, from the transverse part of the duodenum to the transverse mesocolon on the tail side, further right of the duodenal descending limb on the right side, and to the afferent loop for gastrojejunostomy on the left side. Therefore, it does not exactly match the first definition of Behrns et al. However, the fact that the central lesion was the pancreatic head cannot be ruled out based on images. Our case met other definitions.

Pancreatic MALT lymphomas are extremely rare in PPL. In a PubMed search for “pancreas” and “MALT lymphoma,” pancreatic MALT lymphoma was reported in only two cases to date [18, 19] (Table 3).

**Table 3:**
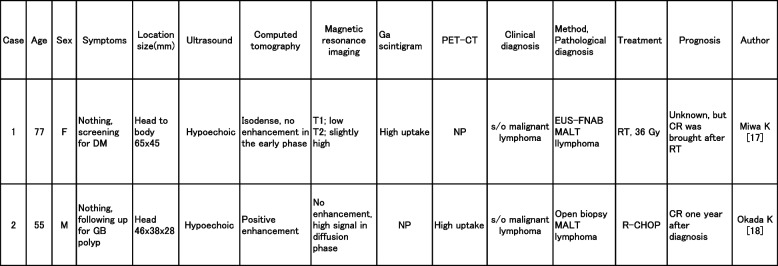
Reported cases of pancreatic MALT lymphoma

Both were asymptomatic, relatively large pancreatic head masses found incidentally during screening for diabetes mellitus and follow-up of gallbladder polyps. Hypoechoic areas were detected in both cases, of which the lesions were not enhanced on contrast CT in Case 1 but Case 2. Case 2 showed high-signal intensity on diffusion-weighted MRI. Ga scintigraphy and FDG-PET showed increased uptake in Cases 1 and 2, respectively. The diagnosis in both cases was malignant lymphoma that was diagnosed as MALT lymphoma using endoscopic ultrasound-guided fine-needle aspiration biopsy and laparotomy biopsy in Cases 1 and 2, respectively. Remissions were achieved by radiotherapy in Case 1 and R-CHOP therapy in Case 2.

Determining the origin of the MALT lymphoma in the present case has been controversial since it was not known whether it originated from the pancreatic head or duodenum. CT and MRI showed a large iso-dense tumorous lesion mainly located in the pancreatic head and expanding to peripancreatic tissues, including the duodenal wall and hepatoduodenal ligament. Therefore, a pancreatic head origin may be possible. On the other hand, histopathological examination of the resected specimen revealed that lymphoepithelial lesions were focally seen in the duodenal mucosa, but not in the pancreas. Moreover, MALT lymphomas usually arise from the mucosa-associated lymphoid tissue of the gastrointestinal tract, and the lymphoma originating from the duodenum can easily invade the fatty tissue of the pancreatic head; thus, a duodenal origin may also be possible. Considering these, the origin of MALT lymphoma in the present case remains unknown.

It is difficult to make a preoperative diagnosis of a collision tumor. In only one of the nine reported cases, a collision tumor of pancreatic ductal adenocarcinoma and NET was diagnosed by preoperative endoscopic ultrasound-guided needle biopsy. In this case, a collision tumor could be suspected because preoperative contrast CT showed distinct characteristics of both tumors, and a definitive pathological diagnosis could be made by preoperative endoscopic ultrasound-guided needle biopsy. Also, in our case, CT, MRI, US, and PET-CT could clearly distinguish heterogeneous tumor lesions, and a preoperative diagnosis of a collision tumor could be made based on percutaneous ultrasound-guided needle biopsy. However, in the other eight cases, collision tumor had not been suspected at all because the two components were not clearly distinguished in preoperative images, or the one element was too small. When obtaining inconsistent findings on CT or MRI for the diagnosis of tumor lesions, we must also consider the presence of a collision tumor. It is also critical to make a definitive diagnosis by needle biopsy when determining the treatment strategy.

## Conclusions

This is the first report of a surgically resected collision tumor of pancreatic ductal adenocarcinoma and MALT lymphoma originating from the peri-pancreatic head. We were able to preoperatively diagnose it using percutaneous echo-guided needle biopsy and multiple imaging modalities, which provided beneficial information in considering the treatment strategy for the tumor. A needle biopsy is useful when inconsistent findings are observed on diagnostic CT and MRI of tumor lesions since there is the possibility of a collision tumor.

## Data Availability

Not applicable.

## Abbreviations

CT: Computed tomography
DWI: Diffusion-weighted imaging
FDG: Fluorodeoxyglucose
GIST: Gastrointestinal stromal tumor
MALT: Mucosa-associated lymphoid tissue
MRI: Magnetic resonance imaging
PET: Positron emission tomography
PD: Pancreaticoduodenectomy
PPL: Primary pancreatic lymphoma
SMA: Superior mesenteric artery
UICC: Union for international cancer control
US: Ultrasonography
